# Towards an Early Warning System for Forecasting Human West Nile Virus Incidence

**DOI:** 10.1371/currents.outbreaks.f0b3978230599a56830ce30cb9ce0500

**Published:** 2014-05-30

**Authors:** Carrie A. Manore, Justin K. Davis, Rebecca C. Christofferson, Dawn M. Wesson, James M. Hyman, Christopher N. Mores

**Affiliations:** Center for Computational Science, Tulane University, New Orleans, Louisiana, USA; School of Public Health and Tropical Medicine, Tulane University, New Orleans, Louisiana, USA; Department of Pathobiological Sciences, Louisiana State University, Baton Rouge, Louisiana, USA; School of Public Health and Tropical Medicine, Tulane University, New Orleans, Louisiana, USA; Department of Mathematics, Tulane University, New Orleans, Louisiana, USA; Department of Pathobiological Sciences, School of Veterinary Medicine, Louisiana State University, Baton Rouge, Louisiana, USA

**Keywords:** arbovirus, disease model, infectious disease, principal components, statistical model, statistics, West Nile virus

## Abstract

We have identified environmental and demographic variables, available in January, that predict the relative magnitude and spatial distribution of West Nile virus (WNV) for the following summer. The yearly magnitude and spatial distribution for WNV incidence in humans in the United States (US) have varied wildly in the past decade. Mosquito control measures are expensive and having better estimates of the expected relative size of a future WNV outbreak can help in planning for the mitigation efforts and costs. West Nile virus is spread primarily between mosquitoes and birds; humans are an incidental host. Previous efforts have demonstrated a strong correlation between environmental factors and the incidence of WNV. A predictive model for human cases must include both the environmental factors for the mosquito-bird epidemic and an anthropological model for the risk of humans being bitten by a mosquito. Using weather data and demographic data available in January for every county in the US, we use logistic regression analysis to predict the probability that the county will have at least one WNV case the following summer. We validate our approach and the spatial and temporal WNV incidence in the US from 2005 to 2013. The methodology was applied to forecast the 2014 WNV incidence in late January 2014. We find the most significant predictors for a county to have a case of WNV to be the mean minimum temperature in January, the deviation of this minimum temperature from the expected minimum temperature, the total population of the county, publicly available samples of local bird populations, and if the county had a case of WNV the previous year.

## Introduction

West Nile virus (WNV) is a mosquito-borne flavivirus first identified in Uganda in 1937. WNV continues to be a public health hazard in the continental United States 13 years after its introduction into New York City in 1999 where it initially caused significant bird mortality and neuroinvasive disease (NID) in humans. In 2012, the USA experienced one of the worst years on record for human WNV 1, with a higher than expected number of 5,674 cases reported to the CDC 2; this is in contrast to the three-year period from 2009 to 2011, during which a total of 3,809 cases were reported. In 2012, 2,873 NID cases were reported from 976 counties across the 48 contiguous states, the District of Colombia and Puerto Rico. There were 270 fatalities among the NID cases 3. Only about 20-30% of the people infected with WNV develop symptoms and less than 1% of people infected develop NID. The states with the highest incidence of WNV in 2012 were North and South Dakota, Louisiana, Texas, and Mississippi, indicating a large geographic range of intense transmission that year 3.

Recent studies indicate that the genetic makeup of the virus isolated from the 2012 outbreak in Dallas is not significantly different from previously circulating isolates, and that all are of the WN02 genotype 4. This suggests that a change in viral efficiency or virulence is not a major contributing factor of the 2012 outbreak. The increased incidence could be caused by extrinsic factors, such as extreme environmental conditions and the potential for these conditions to drive changes in the interaction or coincidence of bird, mosquito, and human populations. Some studies have correlated drought conditions to higher concentrations of mosquitoes and early migration of birds 5. Migratory birds have been implicated as important in the spread of WNV virus 6, while other studies have shown the highest seroprevalence in resident bird species 7 ^,^ 8.

**WNV Transmission Cycle:**
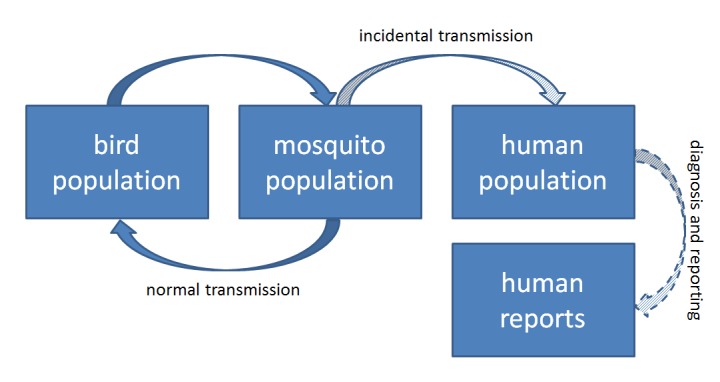
Simplified model of West Nile virus transmission cycle. Human WNV activity is tangential to the enzootic, avian-centric transmission cycle. Reported cases of human WNV represent only a portion of the total transmission in the human population.

The WNV cycle is characterized in Figure 1. Birds and mosquitoes amplify and pass on the virus regularly. Humans are only inoculated by mosquitoes and do not, under normal circumstances, reach high enough viremia levels to pass virus back into the system 9 . Except from the perspective of human health, an examination of infection in the enzootic cycle—birds and associated mosquitoes—is likely more informative than any measure of human cases 10 . This is especially true as mosquitoes can be collected and tested in large batches. Indeed, as WNV spread across the country after introduction in 1999, mosquito abatement and control districts responded by partnering with state and district laboratories to test *Culex spp*. mosquitoes for the presence of the virus. Wild birds are less easily sampled in large numbers for WNV infection and thus mosquito infections and human WNV disease cases are often the only evidence of local WNV transmission.

Despite the availability of these samples, increases in surveillance efforts, and the renewed interest in WNV transmission, reliable predictive methods for human disease outbreaks remain elusive, owing to the milieu of interacting factors affecting overall transmission of WNV. We note specifically the review and novel results of a study in which incidence rates per county in years 2003 to 2008 were characterized by rule-based predictive models conditioned on meteorological and GIS data 11 . The study replicated results from locally conditioned studies and concluded that remotely sensed environmental variables could, with caveats, be used to predict WNV on a national scale.

The study noted a cluster of errors in the Northern Great Plains (NGP) region, which grew in magnitude when a locally conditioned model was attempted 11. We suggest that a study of incidence without reference to measures of healthcare or similar intangibles will be susceptible to systematic biases (e.g. differences in testing and reporting practices) and that this was the case in that study. We note for example that the highest model errors corresponded to the areas of highest unemployment, which is likely a barrier to diagnosis.

The effect of these factors and their interaction on transmission---especially on tangential transmission into human populations—remains poorly understood. We create a predictive framework based on the statistical correlations among:

- the structure of the enzootic host community
- anthropological factors that
  - define differential contact rates of infectious mosquitoes within and tangential to the enzootic transmission cycle
  - influence the probability of diagnosing and reporting human cases
- environmental and weather conditions.

Structure of enzootic host community

**The enzootic cycle of WNV is maintained by avian species and associated mosquitoes, especially *Culex* spp. Transmission characteristics of the avian hosts include host competence variability (relative infectiousness of bird species), susceptibility profiles of the avian communities, and their relative coincidence with human populations 12 ^,^ 13 . For example, small songbirds were found to be highly competent for WNV, indicating their role as likely amplification hosts 14 . Variability of viremia and competence among three bird species was observed in Louisiana, with sparrows having a higher competence for WNV than either cardinals or mockingbirds 15. Also, WNV transmission may be different for migratory and residential bird populations. In India, wild resident water birds were infected with WNV more frequently than wild migratory birds 16. In Virginia, the peak in human incidence occurred after the peak in the enzootic cycle in successive years, indicating the potential for a pattern between the enzootic cycle and tangential transmission into the human population 17. In Los Angeles, a loss of resident avian immunity was associated with human outbreaks 7. We include a broad diversity of bird species in modeling efforts.

Anthropological factors

Contact between infectious mosquitoes and susceptible humans is a function of many factors, several anthropological in nature. Human population density relative to mosquito population size (mosquito density) is included in the measure of arbovirus transmission, vectorial capacity 18. In addition, one of the most mathematically powerful parameters of transmission is the biting rate, a direct estimate of mosquito-to-host contact 19. In the context of human population risk, the likelihood of contact between humans and mosquitoes can be affected by many things, including demographic and socioeconomic factors, as well as the proximity of the enzootic cycle 17. For example, researchers have associated hot spots of WNV transmission with—in addition to ecological parameters—demographic variables, specifically human population characteristics and per capita household income 20 ^,^ 21 ^,^ 22. The age of housing structures and race were significant factors associated with WNV disease in urban Chicago and Detroit 23. In terms of disease reporting as a covariate, a recent study demonstrated that reliance on NID reporting alone did not accurately predict the magnitude of the 2012 outbreak 2. Thus we used all reported cases, including both NID and non-NID.

On a smaller ecological scale, land cover is associated with differences in presumed transmission. In Louisiana, the probability of a mosquito pool being positive is greater in wetland or forested areas than in developed areas 24. In South Dakota, landscape-scale environmental factors are associated differentially with degree of urbanization. In less developed Aberdeen, WNV activity is positively correlated with grass/hay land cover and emergent wetlands while in Sioux Falls, WNV risk is higher in surrounding, relatively rural communities rather than urbanized cityscape 25. Further, agricultural areas are at higher risk in Iowa for WNV than are urbanized areas 26. However, there is no single modeling effort that accounts for the enzootic cycle, its interaction with human population and abiotic factors in order to make national-level predictions about WNV transmission, though subsets of these factors have led to predictive national models. For example, a national study found that the relationship between land use, demography, and WNV incidence vary for different regions of the U.S. as defined by mosquito species distributions 27. Indeed, researchers have asserted that the association of land use and WNV incidence is regionally specific 28, though this model did not make use of weather data as we propose herein. Thus we considered a number of demographic and land use variables in our national model.

Environmental and weather conditions

Arbovirus transmission is altered by several environmental and weather conditions. For example, temperature is a known modifier of WNV efficiency, directly reducing the time necessary for the mosquito to become infectious after exposure or increasing the number of mosquitoes ultimately infectious 30 ^,^ 31 ^,^ 32. Further, WNV incidence in Georgia was associated with increases in minimum temperature early in the calendar year 33. Recently, drought conditions (increased temperature, decreased precipitation) were positively associated with increases in WNV transmission 5 ^,^ 34. Other studies have shown the positive correlation between WNV transmission and temperature, as well as an inverse or no relation between WNV and precipitation levels 35 ^,^ 36 ^,^ 37 ^,^ 38 ^,^ 39 ^,^ 40. While other environmental conditions (or different measures thereof 36) may contribute to changes in transmission, temperature and precipitation are by far the most commonly used abiotic metrics.

Model Rationale

Anecdotal evidence from mosquito abatement experts suggests that drought conditions may have contributed to the increase in human WNV activity in 2012, with published research supporting this hypothesis 5. Our goal was to determine if there are predictive variables for the risk for increased human WNV infections early enough to support potential large scale (regional or national) preparedness. As mentioned above, a study in Georgia associated WNV transmission with minimum temperature in January for 2002-2004 33 and high temperatures in winter were associated with the 2012 Dallas outbreak 40. Preliminary investigation of this relationship on the national scale and over several years (2005-2013) showed a continued strong correlation between the deviation from usual minimum temperature in January and reports of WNV in that year (Figure 2).

**Minimum temperature predicts reporting:**
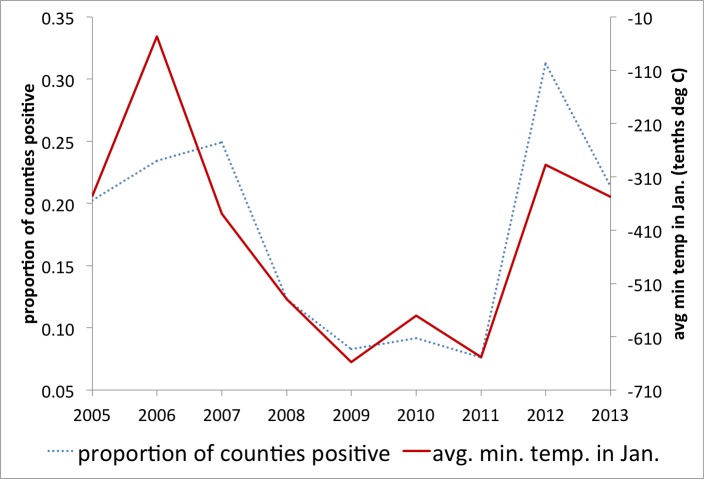
Relationship between the mean minimum temperature in January and the proportion of counties reporting West Nile Virus later that same year.

Thus, we continued this line of investigation and included several variables related to the enzootic cycle, human demographic data, and other environmental variables. The study relies on historical and publicly available data sets that are available early in the year in question (including the previous year’s WNV report, remote sensing data, environmental parameters, etc.), so that the model may be employed as an early warning system and results used to support policy decisions and resource allocation.

## Methods

We developed a statistical model to predict yearly national human WNV risk with data collected early in the year and available for all counties in the contiguous United States. We hypothesized that although risk factors vary by region, deviation from minimum temperature in the winter is associated with WNV risk across regions. Based on previous studies and the ecology of WNV, we included multiple demographic, ecological, and climatological variables to build our statistical model with the goal of finding a simple cohort of variables that accurately predict increases in human WNV risk.

National geography and WNV reporting 

We obtained county boundaries for the 48 contiguous US states from the SAS base maps file (SAS 9.3, Cary, NC). The coordinates of the county centroids were approximated by the mean of points on the boundary of each county. Cumulative yearly counts of all reported WNV cases in humans reported to the CDC by county were obtained from the USGS summary for 3,111 counties from 2005 to 2013 41.

We believe that attempts to characterize incidence within a county expose the model to the problem of small numbers. In this case, the number of samples (the population) within a county is usually large and the number of positives is relatively small. Young et al. discusses three methods to address the problem: spatial smoothing, aggregation over geographic areas, or aggregation over time 11. As in that study, we take the county and year as our basic units.

The Young et al. study also concluded that their attempt had not mitigated the small numbers problem. While we suspect, as the authors did, that confounding factors were at least partially responsible, we also suspect that characterization of incidence by incidence rate and multivariate linear models is likely inappropriate 11 . We follow the arguments of O’Hara and Kotze (2010) in which it is argued that count data are best modeled by Poisson or negative binomial distributions, rather than by transformation and approximation by a normal random variable 42. This is especially true in the current data, with the majority of the reported rates being zero.

Here, if a county had reported any human WNV cases, it was coded as 1 and 0 otherwise. We argue that variations in this (0/1) metric capture the majority of variation in the total number of WNV cases across space and time and a more detailed model is not currently indicated (e.g. see Figure 3). This dichotomous method has been successfully employed at a regional level 43.

**WNV human cases and reporting per county:**
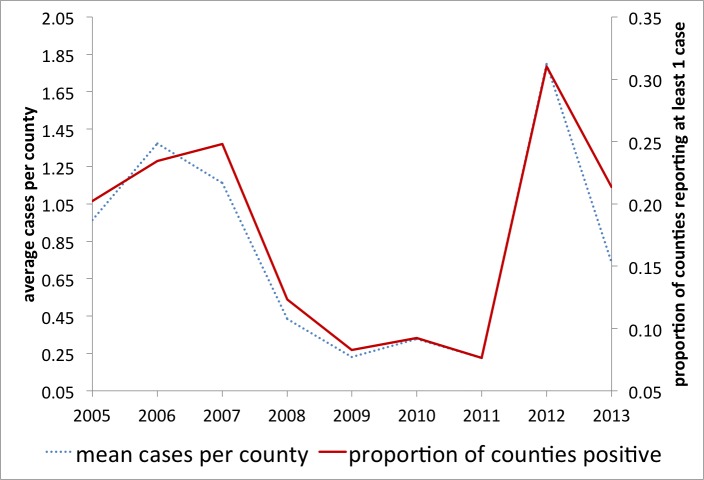
Yearly comparison of the average cases of WNV per county (total WNV/counties reporting) to the proportion of counties reporting at least one case of WNV that year, 2005-2013.

Covariates

Bird community. We used the Breeding Bird Survey (BBS) to approximate numbers of birds, summarized by order, in an area. Surveys are comprised of counts made along distinct 24.5-mile routes in .5-mile intervals. Each survey was summarized by year conducted, route sampled, and order of bird species identified. Twenty-seven orders were represented and zeros were imputed when no examples of an order were reported along a particular survey route. Two attempts were made to reduce the dimensionality of the set. First, the total number of birds identified in a survey was log-transformed; this correlated poorly with WNV reports and was not further investigated. Next, log-transformed counts by order were subjected to a principal component analysis; the principal components led to a relatively compact description of the BBS. The principal components were computed for each year per route and represent a linear combination of the log (order count) for orders 1-27.

It is important to note that the BBS data set was included here not so that individual species or orders could be associated with WNV, but as a means to account for unique regional ecologies and bird community biodiversity. Thus, the parameter estimates for principal components do not correspond to any specific characteristic of the bird community and are meant as markers for underlying environmental intangibles.

We extrapolated the set of principal components to county centroids with a power semivariogram model (proc variogram and proc krige2d in SAS 9.3) (SAS®, Cary, NC). Estimates were conditioned on the nearest 50 points to avoid prohibitive computational complexity. Similar semivariogram models were considered and most yielded qualitatively similar results; the power model was chosen for its smaller number of parameters.

Anthropological data. We used the 2010 census demographic data on a per-county basis. The data included estimates of the total population, median total income, proportion of the population in the labor force, and proportion of the population by race. The first two variables were log transformed and log-population was included as a second-degree polynomial (the quadratic form has been used successfully in other local studies 33). A nonlinear relationship was observed in the marginal distribution, with low and high population counties more frequently reporting WNV than mid-size counties. Measures of density, e.g. persons per square mile, did not improve on the models and were excluded from consideration.

The National Land Cover Database 2006 (NLCD2006) from the Multi-Resolution Land Characteristics Consortium (MRLC) classifies land areas into one of 16 categories. We summarized each county by the proportion of pixels within its boundary classified as developed (types 21-24) in Quantum GIS (QGIS).

Finally, we investigated the potential for county-level reporting biases by including WNV reporting in the previous year and, in separate models, the previous two years. We reasoned that biases not otherwise addressed might be corrected by continued deviation from expectation. Both year and previous two years were significant predictors of WNV status (p<.05) but were also highly correlated. In the final model, we determined that the previous year’s report was sufficient.

Environment and weather. Our models initially included maximum temperature, minimum temperature, precipitation, and deviation from the average for each of these variables. While the WNV cycle depends on covariates throughout the year, mean minimum temperatures in January alone were retained from the Global Historical Climatology Network monthly summary (GHCN-M) for all available stations; stations with any missing values in January 2005 to 2011 were excluded. Expected mean minimum temperatures for a station for January were defined as the average of that month’s data from 2005 to 2011. Various periods were considered (e.g. 1970 to present) to define the expected temperatures but yielded no qualitative differences, indicating that 2005 to 2011 was in some way representative. These indices were extrapolated to county centroids by weighing the nearest 50 stations by distance from the centroid.

Maximum and average temperatures were individually significant predictors, but were not as powerful as minimum temperature and deviation from minimum temperature and did not improve on models already containing minimum temperature. We made an attempt to include precipitation, but the semivariogram fits were poor and extrapolation to county centroids was questionable. This seemed to indicate that precipitation was a local phenomenon with scale smaller than we were able to observe. Thus maximum temperature, average temperature, and precipitation are excluded from the final model.

Statistical analysis

We carried out logistic regression analyses in SAS 9.3 using the dichotomous (0/1) variable indicating WNV activity for each county-year. A stepwise procedure with entry/exit p < 0.10 (proc logistic) was used to determine which variables best fit the data. We sought influential observations in the selected model by DFBETA; exclusion of the most influential observations produced no qualitative changes and no observations were deemed outliers.

Because we repeatedly sampled the same counties over time, we do not model all samples as independent. Two samples from the same county were assumed to correlate even after the influence of all covariates had been removed. In the final model, we employed a generalized estimating equation (GEE) regression with an exchangeable working correlation structure (proc genmod in SAS 9.3) and estimated this correlation coefficient as ρ=.1041 for 2005-2011 data. This indicates that when modeled effects are considered, the remaining, unexplained correlation among WNV reports within a county is not prohibitively large. Regular and standardized regression coefficients and chi-square statistics were calculated for the resulting model.

## Results

We recall that the model is conditioned on WNV human reported incidence data from 2005-2011 and we generated predictions for 2012-2013 without training on those years. The area under the receiver operating characteristic curve (AUC ROC or *c*) and the Hosmer-Lemeshow test statistic were calculated to measure the fit of the model for the 2012 data 44.

The predictors included in the final model for 2005-2011 (Table 1) are ranked by the magnitude of the standardized parameter estimate, in which larger values indicate more sensitivity of WNV status to changes in the covariate. The five most powerful predictors of WNV reporting in a county in order are

1. the expected mean minimum temperature in January
2. the actual mean minimum temperatures in January
3. the total population of the county per the 2010 census
4. the first principal component of bird data
5. the WNV status of the county in the previous year

In 5,000 simulated case-control studies, in which 25% of all positive and 25% of negative counties from years 2005-2012 were randomly chosen for training, the order of these components was unchanged in only 21% of trials. When population is omitted, this rose to 98% and none of the coefficient estimates were ever observed to change sign. This indicates that population is primarily important in differentiating relatively large, rare counties, which might not be present in random samples, from smaller, more numerous counties. This also indicates that the primary hypothesis, dependence on mean and actual temperature in January, is robust against missing data and selection of different training sets.

**Model for predicting human WNV activity:** Model variables and statistical summary for prediction of human WNV activity, 2005-2011. "BP" = Bird Principal component for a particular year.

parameter	estimate (log odds)	standardized estimate (c/σ)	std. error	p>Χ^2^
intercept	-0.547		1.4253	0.7012
expected mean minimum temperature in January	-0.00224	-0.6185	0.000107	<0.0001
mean minimum temperature in January	0.00185	0.5157	0.00008	<0.0001
(log total population)^2^	0.0284	0.4673	0.00113	<0.0001
first principal component of bird data (BP1)	0.2714	0.3615	0.0164	<0.0001
WNV present in previous year? (Yes=1)	1.0758	0.2606	0.0503	<0.0001
BP3	-0.244	-0.2365	0.0166	<0.0001
BP2	0.1981	0.1810	0.0208	<0.0001
% Black or African American	0.0144	0.1372	0.0021	<0.0001
BP20	0.4544	0.1130	0.0512	<0.0001
BP12	0.0751	0.1043	0.0379	0.0475
BP18	-0.3397	-0.0886	0.0518	<0.0001
BP5	-0.2767	-0.0807	0.0242	<0.0001
BP7	-0.1660	-0.0745	0.0292	<0.0001
% employed (Census 2010)	2.1885	0.0686	0.4636	<0.0001
(proportion developed)^2^	1.278	0.0667	0.5282	0.0155
log (total income and benefits)	-0.6033	-0.0623	0.1488	<0.0001
BP6	0.1508	0.0556	0.0208	<0.0001
BP16	-0.1021	-0.0500	0.0453	0.0240
proportion of county land developed	-0.7137	-0.0454	0.2931	0.0149
BP13	-0.2086	-0.0366	0.0360	<0.0001
BP10	-0.1220	-0.0342	0.0314	0.0001
BP15	-0.0794	-0.00937	0.0430	0.0649
BP19	-0.1294	-0.00609	0.0525	0.0137
BP21	0.2658	0.0045	0.0603	<0.0001
BP9	-0.1046	-0.0042	0.0348	0.0027

Additionally, WNV is positively associated with employment levels in a county while actual income levels are negatively associated with WNV reported human cases. The percent of African Americans in a county is also a positive predictor for WNV reporting.

Observed and estimated proportion of counties reporting positive per year are shown in Figure 4. When conditioned on 2005-2011 reported human WNV cases, the model prediction for 2012 had c-value of .849, which Hosmer and Lemeshow consider to show excellent but not outstanding discrimination 44. In the Hosmer and Lemeshow goodness-of-fit test, the model was well-calibrated at the 10% (but not 5%) level of significance (p = .0780).

**Estimated rate of WNV human activity:**
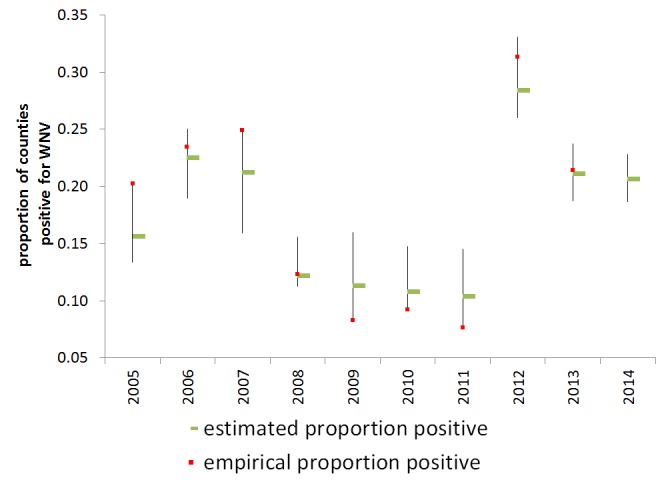
The empirical (red square) and estimated (green bar) rate of presence of WNV activity with the 95% confidence intervals of the statistical prediction.

We present the empirical and estimated rates of positivity for 2013, but note that the empirical proportion is likely an underestimate, as some states are at the time of writing only reporting on a state-wide level and not all reporting has been completed. Data in 2013 were used neither to condition nor to validate the model. So far, 2,374 human cases were reported in 2013 with 1,205 NID cases reported in the United States. The Hosmer and Lemeshow test for 2013 is well calibrated at the 10% level of significance (p=0.0818) . We also present predictions for 2014 based on the available preliminary 2013 WNV human data (missing 2013 county-level data was replaced with 2012 data) and temperature data for January 2014.

## Predictions for WNV in 2014

The unusual distribution of temperatures in the 2013-2014 winter gives a rare opportunity to further investigate the link between temperature and human WNV. Approximately half of the country (east) experienced record low temperatures while the other half (west) experienced milder, even unusually warm temperatures, as in Figure 5.

In Figure 6, we provide the predicted probability of reporting at least one case of human WNV, binned into low, medium, and high risk classes (lowest 60% of counties, 60-85, and 85+ respectively). We predict that there will be more WNV infections than usual in the western US and fewer than usual in the eastern US. However, the total number of positive counties in 2014 is predicted to be comparable to the total in 2013, as nationwide average temperatures were comparable between these two years. Therefore, we encourage increased vigilance in western counties where we predict an increased risk of human WNV cases.

**Deviation from expected temperatures in Jan. 2014:**
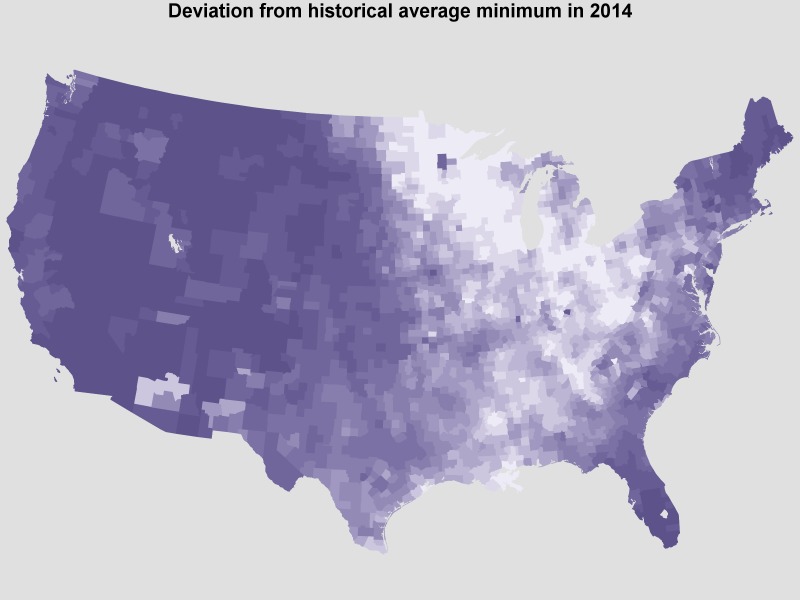
Average of daily deviation from historical minimum temperatures in Jan. 2014 by county, darker = warmer.

**Predicted probability of county eventually positive for human WNV in 2014:**
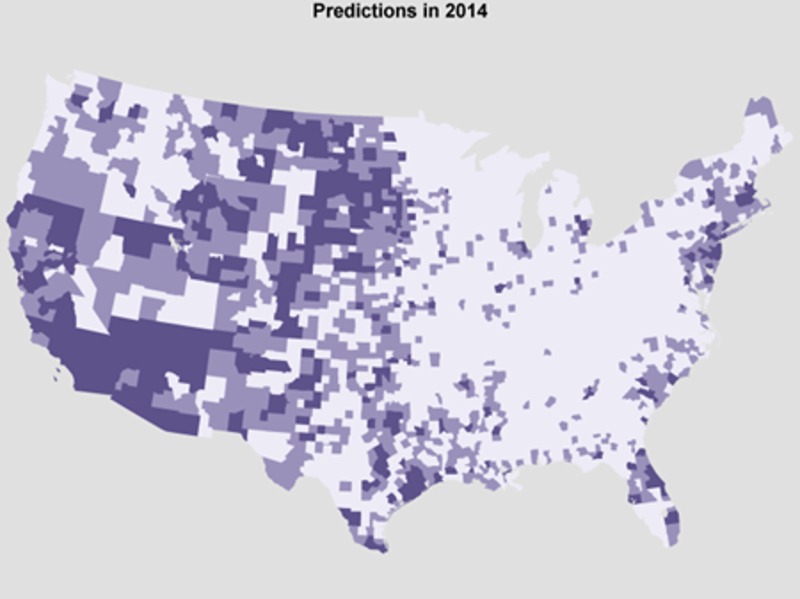
Predicted probability of reporting at least one human WNV case in 2014, binned by threat category, darker = more likely to report positive.

## Discussion and Conclusion

Several modeling, laboratory and field studies have shown that WNV transmission is associated with environmental factors such as temperature, precipitation, drought, and land use, as well as biological factors such as bird community structure and anthropological variables, including urbanization and human density 8 ^,^ 45. However, most of the studies focused exclusively on particular states, counties, or regions based on the data available to the researchers. There is a need for a concise, relatively simple model that can predict early in the year when risk of human WNV may be elevated in order to inform public health decisions, resource allocation, and public education.

By focusing on the development of an early warning system from a national perspective, we directly speak to this need for better predictive tools to guide decisions regarding control and mitigation. Our model shows that in the contiguous United States, bird presence estimated from the previous year, deviation from minimum temperature in January of the year in question, human density estimates from census data, and history of WNV reporting are sufficient for prediction of risk of WNV human activity later in the year in question.

In fact, conditioned on the 2005-2011 data, our model predicted the large upswing in human WNV in 2012, requiring only data from the previous year and temperature data from January 2012. To the extent that the model holds, none of the years in question are anomalous (Figure 4).

Specifically, we found that the most significant predictors of WNV were the expected and actual minimum temperatures in January of the year in question. These do not seem to admit interpretation as biases and likely have straightforward biological interpretations. For example, unusual, positive deviations in temperature early in the year may increase a mosquito’s probability of surviving the remainder of the winter, may shorten the extrinsic incubation period, or encourage earlier and higher rates of mosquito breeding, and have been associated with heightened bird activity such as greater numbers of resident birds nesting and migration of non-resident birds 31 ^,^ 38 ^,^ 40. Deviation from minimum temperature accounts for relative variations in climate and for local adaptations of mosquitoes and birds to their environment, which actual minimum temperature alone cannot capture.

Higher county-level income measures are negatively associated with reported WNV human incidence. This is supported by several studies, which have shown that higher housing density (as seen in lower socio-economic statuses) and increased urbanization are positively associated with WNV in humans 46 ^,^ 47. Further, the proportion of African Americans in a population was a positive indicator of human WNV activity. The significance and interpretation of this factor is likely regionally specific and requires a more anthropological and socio-economically targeted study to determine context.

The dual importance of human density and general bird presence is intuitive, given that the spillover into humans requires proximity to the otherwise bird-driven WNV transmission cycle. Again, specific orders of birds or specific species composition could be important on a local or regional level. In this model, however, bird orders serve more as proxies for the entirety of the underlying system and do not admit direct interpretation of the specific makeup of avian communities.

The idea of birds as proxies is supported by studies that have shown different bird species to have higher seroprevalence than others (e.g. northern cardinal in Georgia 48, robins 49 , house finch and house sparrow in California 7). Likely, regional iterations of this model will show more consistency with regard to specifics about the bird community, as this is ecologically driven, although the results of Young et al. call into question this intuition 11.

This model can be used as an additional tool for public health institutions in the United States, as a national indication of the potential for human WNV activity increases, and eventually at the regional or local level, to inform surveillance, mitigation, and resource allocation efforts. For example, once on high alert, counties or regions can consider surveillance data, specific climate data, mosquito ecology for the region, land use, bird ecology, etc. and develop models such as ours for their particular regions, allowing them to continue to monitor risk and implement mitigation in a timely manner. In future work, this model will be applied regionally to show how particular regions can use additional weather data (precipitation, soil moisture levels, etc.), land use data, bird data and mosquito surveillance to determine further risk that year.

The model uses data from the first month of the year in an effort to provide some confidence in its performance going forward and to provide earliest possible warning of heightened WNV risk. Since this model is based in reported human cases, its predictive capability is limited to just that. Thus, it should not be used to make any assumptions about the total volume of virus in the enzootic cycle, or the true transmission intensity, as most human cases are asymptomatic. This model is proposed on a national scale, and the intention of its use is to raise awareness of a potential heightened WNV season in time for any resulting reactions to have transmission mitigation efficacy. We envision that this model will be the first step in a series of spatially tiered warning systems that will increase in regional specificity and timing.

Since the primary vectors of WNV are regionally dependent and given the spotty nature of consistent county-level mosquito control and surveillance data, mosquitoes were not explicitly considered in this model. Additionally, it is difficult to quantify the impact of mitigation strategies such as spraying on human incidence over multiple years without bird and mosquito surveillance data. This speaks to the lack of consistent data of both bird and mosquito populations in many regions, which would improve the predictive capabilities of any model, the regional scale at which a model could be formulated, and our understanding of WNV dynamics. On county and regional levels, models with access to detailed bird and mosquito surveillance data have had success in explaining WNV incidence in the enzootic and tangential cycles based on ecological and environmental factors 5 ^,^ 7 ^,^ 22 ^,^ 31 ^,^ 33 ^,^ 37 ^,^ 40 ^,^ 45 ^,^ 50 ^,^ 51 ^,^ 52. Delving into the specifics of regional climates, and into the social and land planning factors that increase risk will also be important for future efforts. Bird diversity can also be an important factor in national WNV spread and human risk 53. Additional data sources (seroprevalence from bird populations, heterogeneity of infectiousness among bird populations, the sero-conversation rate of sentinel chickens, and mosquito populations) are needed to truly understand the transmission cycle of WNV. This understanding would lead to better predictions of transmission intensity and thus inform mitigation strategies and ultimately reduce human cases. However, in the absence of these explicit models, our model is a tool for early warning and policy decision support.
